# Disaster preparedness training for Latino migrant and seasonal farm workers in communities where they work

**DOI:** 10.1186/s12995-018-0219-4

**Published:** 2018-12-17

**Authors:** Rene P. Rosenbaum, Brenda Long

**Affiliations:** 1 School of Planning Design and Construction, Michigan State University, Human Ecology Building, 552 West Circle Drive, Room 214, East Lansing, MI 48824 USA; 2 Michigan State University Extension, East Lansing, USA

## Background

Hispanic migrant seasonal farmworkers (MSFWs) are essential to the sustainability of many commercial fruit and vegetable farm operations in rural counties across Michigan as well as those in many other states across the nation [1]. Migrant farmworkers usually travel long distances from their sending communities in southern states, like Texas and Florida, to work field agriculture, nurseries, greenhouses, and processing plants in receiving communities of the north, states like Michigan and Wisconsin, during the summer and fall harvest seasons. Although no current data system provides a reliable count of the migrant farmworker population, the population has leveled off since the 1990s when roughly 2.5 million farmworkers were employed in the U.S. Migrant farmworker expert, Philip Martin, estimated 840,000 farmworkers have 409,000 children traveling with them as they do farm work [2].

Climate change and the increase in sudden and destructive and, at times, catastrophic events have made MSFWs in the United States increasingly vulnerable to disasters, more so than the general population [3, 4]. MSFWs and their families face unique issues during disasters (e.g., natural, chemical, biological, man-made disasters) that can result in death or significant injuries. Like other immigrant groups in poverty, they often lack proper resources for emergencies and face transportation and language barriers. Farmworkers are susceptible to crop and animal disease outbreaks on the farms where they work and where they and their families often live. Due to the migratory nature of their work, often they are not familiar with the receiving communities where they travel to. That is particularly the case for the many farmworkers who live in agricultural labor camps that are typically located in remote rural areas. Additionally, this hard-to-reach population often lacks transportation and faces language and cultural barriers in accessing the public health protections available. While the latter barriers exist during normal times, in times of disaster-related emergencies and crises, they can become a matter of life or death.

MSFWs are also at a significant risk of not being adequately prepared to respond to disaster emergencies. The Federal Emergency Management Agency (FEMA) has offered free disaster preparedness training for community volunteers across the country since 1993, but not specifically for Hispanic MSFWs. Even if training is available, finding the time to train in disaster preparedness is a challenge for farmworkers who often travel long distances to work in communities away from their homes. Additionally, farmworkers do not often integrate well into their receiving communities, rendering this population less likely to receive the emergency responsiveness training provided to local residents. What emergency preparedness training is available in receiving communities is not likely to be culturally or linguistically appropriate. The fact that migrant farmworkers are often away from their home base also makes it challenging for this occupational group to take advantage of the emergency preparedness training opportunities available in their sending communities.

While there is increasing documentation and awareness of the high risk Hispanic MSFWs face during disasters, the emergency training approaches specific to migrant farmworkers are limited. Materials targeting the training needs of urban immigrant and linguistically isolated populations are increasingly given a Spanish flavor; however, only two disaster preparedness training approaches specific to migrant farmworkers were identified from a literature review on the subject. Moreover, neither of these approaches has targeted the farmworker population directly, but rather has targeted the training needs of public health professionals [5, 3]. A third approach, concerned with the disaster preparedness needs of “newcomers,” a collective term that also includes migrants, targets the training needs of first responders to enable them to deal with newcomers in the event of a disaster [6].

This manuscript reports on an emergency preparedness training approach that directly targets the MSFW population. The report describes the planning, implementation, and assessment of the Migrant and Seasonal Farmworker Disaster Preparedness Demonstration (MSFW-DPD) project, a 20-h long educational intervention aimed at closing the gap that exists in disaster preparedness and in disaster preparation training in the MSFW population. The study illustrates how a University partnership involving the Extension Service,  providers of MSFW human services, and county emergency services proved essential in overcoming barriers associated with emergency training to the MSFW population when they are in receiving communities where they travel to work.

The MSFW-DPD project relied on the Community Emergency Response Team (CERT) curriculum (< https://www.ready.gov/community-emergency-response-team >) for its training. CERT is a national program developed by the Department of Homeland Security through FEMA to educate people about disaster preparedness for hazards that may affect their areas [7]. In this training, participants learn readiness and response skills they can use to help others in the event of a disaster and serve a vital role in the interest of public health prior to the arrival of first responders and can assist these responders when they arrive. The CERT programs provide national preparedness guidelines for training, a factor deemed important given the multistate travel patterns of the migrant farmworker population.

### Goal and objectives

Across the country, local CERT programs provide training and support for teams in all sectors of the community – neighborhood residents, employees, and students in high school, college, and many other groups. However, to date, this training has not extended to the MSFW population. The purpose of the MSFW-DPD project was to design and deliver disaster preparedness training to Hispanic farmworker youth and adults, as well as to demonstrate the program’s feasibility and effectiveness and to document lessons learned. The overall objective of the training component of this program was to increase awareness and build capacity in the farmworkers’ ability to respond in their communities in the event of a disaster.

The project had four process objectives. The first was planning the design and implementation of a disaster preparedness program. A second objective was to promote the training program in the farmworker community and to identify, recruit, and select training participants. The third objective was to use the CERT program to educate forty Hispanic farmworker youth and adults on disaster preparedness for hazards that could affect them where they were located. The final objective was to disseminate the results of the study through various venues, including the production of YouTube videos featuring the MSFW children and adults that participated in the training.

### Target audiences

The last census of MSFWs in the State of Michigan was in 2013 [8]. That report concluded the MSFW population in the state included 49,135 farmworkers and 45,032 other persons accompanying them. Of these, 42,729 were children and youth under 20, of which 35% were between the age of 13 and 19.

The target population for this training demonstration was the 6960-estimated MSFWs in Oceana County. As the third largest user of MSFWs in Michigan, Oceana County accounts for over 7% of the state’s overall MSFW population. Our calculations suggest over 1000 MSFW youth between the ages of 13 and 19 are in Oceana County each summer. Many of the migrant farmworker families come from Texas and Florida and other states, as well as Mexico. The majority of the population has a low level of education and prefers to communicate in Spanish^1^. Our educational target goal was to train 40 farmworkers on disaster preparedness, with the goal of having both youth and adult participants.

## Methods

The project team conducted two disaster preparedness workshops under the project grant, one in June and the other in October 2016. Initially, the plans called for conducting only one workshop, but the weather influenced the crop harvest and work patterns and, hence, the June workshop attendance. The project team adapted to the situation by scheduling another workshop for the end of the harvest season. Each workshop was structured as a 3-day training program. For the June workshop, the participants spent the night at the facility where the training was taking place, and so were able to work after dinner. For the October workshop, participants went home each evening and returned the next day. These participants received a $20.00 gas gift certificate to cover these costs.

CERT training courses provide workshop participants the opportunity to train in emergency preparedness and basic responses to emergencies. The workshops are intended to ensure that participants have the skills to protect themselves and to assist others in the event of an emergency and before first responders can arrive on the scene. These courses rely on a certified trainer and a team of first responders who have the requisite knowledge and skills to instruct the sessions. The CERT curriculum trains community members in basic disaster response skills such as fire safety, light search and rescue, team organization, incident comment, and disaster medical operations. The training also provides opportunities for participants to practice what they learn by engaging them in disaster simulation exercises. A description of the content of each unit in the CERT program, listed in the order they were taught, follows.

### Unit content

Unit 1: Disaster Preparedness.

This unit instructs workshop participants how to prepare themselves and their community for the various hazards that could occur. Content topics include:

- Roles and responsibilities for community preparedness
- Elements of disasters and their impact on the infrastructure
- Personal and organizational preparedness
- Role of CERTs

Unit 6: Organization and Incident Command

This unit addresses CERT organization and management principles necessary for a CERT to operate successfully. The objectives of this unit were to:

- Describe the CERT structure
- Identify how a CERT interrelates with the Incident Command System
- Explain the need to provide a command post with information and documentation requirements

Unit 8: Scene Awareness and Terrorism.

This unit addresses the dos and don’ts during a terrorist act and provides homeland defense tips. Unit objectives included training workshop participants to:

- Define terrorism
- Identify potential targets in the community
- Identify the eight signs of terrorism
- Identify CERT operating procedures for a terrorist incident
- Describe the actions to take following a suspected terrorist incident

Unit 2 Fire Safety.

This unit covers fire chemistry, fire hazards, fire suppression strategies. Specific topics included:

- Fire chemistry
- Fire and utility hazards in the home, workplace, and neighborhood
- Important role CERTs play in fire safety
- CERT size-up
- Fire size-up considerations
- Firefighting resources
- Fire suppression safety
- Hazardous materials

Units 3 and 4, Disaster Medical Operations, Parts 1and 2.

Part 1enables participants to practice diagnosing and treating airway obstructions, bleeding, and shock by using simple triage and rapid treatment techniques. They engage in event simulations to practice these techniques as they are used to manage mass casualty events. Part 2 covers evaluating patients, establishing a medical treatment area, and performing basic first aid.

Unit 5 Light Search and Rescue.

Unit participants learn the goals of light search and rescue, factors to consider in deciding to attempt rescue, light search and rescue planning, techniques, and rescuer safety. Unit objectives included training workshop participants to:

- Identify size-up requirements
- Describe most common search techniques
- Use safe techniques for debris removal
- Use safe techniques for survivor extrication
- Describe ways to protect rescuers

Unit 7 Disaster Psychology.

This unit covers signs and symptoms that disaster victims and workers might experience. It describes the disaster and post-disaster emotional environment for survivors and rescuers, and the steps rescuers can take to relieve their own stress and that of other survivors. Unit topics include:

- Disaster psychological trauma
- Team well-being
- Working with survivors’ trauma

Money Management, Cardiopulmonary Resuscitation (CPR), and Automated External Defibrillator (AED) Training and Certification.

In addition to the FEMA CERT training course materials, the MSFW-DPD project course includes a session on how to prepare for and manage money in event of disasters. CPR/AED training and certification using *Heartsaver* materials are also covered. The course teaches the critical skills needed to respond to an emergency until emergency medical services arrive. The skills covered in the course include first aid and choking relief for adults, children, and infants. Also covered is cardiac arrest training for adults, children, and infants.

Michigan State University (MSU) and Newaygo County Emergency Services (NCES) instituted a professional services contract on February 28, 2016. The Director of NCES, Jane Peterson,^2^ agreed to consultation with project team leaders to adapt and deliver the CERT training curriculum to migrant and seasonal Hispanic farmworker youth and adults in June of 2016. She also agreed to conduct a similar workshop in October. She met all the deliverables of the agreements between February 28 and October 29, 2016.

### Planning and design

The project team leaders sought stakeholder input from the start. During the proposal-writing phase, they discussed their ideas for the project with a former farmworker and veteran Mason-Oceana County Michigan Department of Human Services (MDHHS) employee who for over 20 years has provided human services to the MSFW population in the area. They also shared their ideas with the chair of the West Michigan Migrant Resource Council, an organization made up of representatives from all the agencies in the area that serve the area’s MSFW population.

The MSFW-DPD project team leaders received notification of the award in the fall of 2015. Because the migrant population did not arrive in the area until the following spring, the project team had about five months to prepare and plan the training program. Planning tasks included identifying curriculum materials, training session planning, translation of forms and procedures, program schedules, and entering into professional service agreements with the CERT trainer, the video producer, and the managers of the facilities we rented for the training. MSU also entered into a Memorandum of Understanding with the MDHHS. The memorandum enabled the Mason-Oceana MDHHS office staff to serve on the project work team and to recruit their MSFW clients to participate in the workshop training.

Although we knew the CERT curriculum was available in Spanish, we discovered during the proposal-writing phase of the project that a bilingual certified CERT trainer was not available in the area. Because many farmworkers are Spanish-only workers, we planned to recruit farmworker youth, who may have been more likely to know English. They were to serve as language and culture bridges and help Spanish-only workers and their parents better understand the material during training sessions. At the same time, however, we arranged to have additional Spanish language facilitators.

### Curriculum development

The training modules developed were essentially the same in both workshops, as the NCES Director applied the same CERT guidelines. Ms. Peterson was consultant on the project and the main curriculum specialist. She took the lead in organizing the training modules and delivered the core curriculum. Learning objectives targeted CERT core competencies. The workshop training sessions followed the CERT training guidelines with attention given to the risk of disasters in the local area. The trainer drew upon a variety of federal, state, and locally developed materials that were adapted to the local area need. The First Aid, CPR, and AED components of the course relied on the American Heart Association’s (AHA) research-proven techniques that allowed instructors to observe the students, provide them feedback, and guide their learning of the relevant skills. The training course provided workshop participants many opportunities to practice emergency preparedness and basic response techniques.

### Coordination and implementation

MSU entered into an agreement with the Director of NCES in February 2016. In addition to working with Peterson to develop the curriculum and deliver the training, the project team made the decision early on in the planning phase of the project to seek cooperation from the staff in the MDHHS at the Mason-Oceana County office. In April of that year, the project team signed a Memorandum of Understanding with the MDHHS. The latter agreement enabled the Mason-Oceana DHHS office staff to work on recruiting MSFWs for the disaster preparedness workshops. These workers had frequent contact with the MSFW population and many were clients they assisted in determining their eligibility for state and federal health and human service programs. Because transportation is often an issue for many farmworkers, the MDHHS staff also conducted outreach and traveled to the various camps to identify farmworkers eligible for their programs. Thus, seeking the cooperation of the MDHHS was important to ensure the Mason-Oceana office staff were involved in recruiting workshop participants from both labor camps and the city. They were also crucial in ensuring the involvement of key partners with strong ties to the Latino farmworker community, such as the Oceana Hispanic Center and the Northwest Michigan Health Services clinic in Shelby, Michigan.

To complement these developments, the project team formed a working group consisting of Extension personnel, MDHHS administration and staff, and the emergency services directors from Oceana and Newaygo counties. Our charge was to design, implement, monitor, and evaluate the project. Over the course of seven months, leading up to the first workshop in June, the project team met nine times at the project location site, which was 150 miles away from MSU. The project team worked extensively with the staff from MDHHS to plan the logistics for the training. Activities included locating a venue; signing service contracts and memoranda of understanding; translating the recruitment materials, including participant and parent consent forms and YouTube photo releases; translating the evaluation forms; marketing the training; recruiting participants, and working with the MDHHS recruiters to track recruitment progress and address recruitment issues. We scheduled the first workshop for June 9, 10, and 11th because those dates coincided with the end of the asparagus season and were considered a good time for the training because the farmworkers had down time. MDHHS staff recruited 38 farmworkers to participate in the training for that day. However, only 14 farmworkers attended the training. Three MDHHS farmworker specialists and four Extension personnel also participated in the training. The relative low turnout prompted the decision to conduct another training session at the end of the apple harvest season in October. Of the eighteen farmworkers recruited for that workshop, nine were able to complete the program. In the end, 30 participants participated in the two workshops. Twenty-three were farmworkers. Both training workshops featured a main trainer and a fleet of 10–12 first responder volunteers who aided the trainer with the disaster simulations. Both workshops also featured Spanish translation and childcare services.

### Key accomplishments

This training project was a collaborative effort between MSU faculty and Extension educators and the MDHHS and NCES. The National Institute of Food and Agriculture (NIFA) provided funding for the project. In preparation for the training project, MSU signed a memorandum of understanding with the MDHHS that granted the personnel in its Mason-Oceana County office to assist with the project, particularly in the area of recruitment. A FEMA certified trainer was identified and an agreement was entered into for the development and delivery of a 20-h disaster preparedness program following the CERT core content areas. Additionally, the team leaders formed a working group to design, implement, and guide the project.

The work group that formed to guide the project met in Oceana County more than 10 times over the course of planning the project, conducting the training, and doing related fieldwork. Members of the workgroup consisted of Extension personnel, MDHHS administration and staff, and emergency services directors from Oceana and Newaygo counties. Staff from the MDHHS Mason-Oceana County office shared information about the training with the clients they sought to recruit. As part of the recruitment effort, a letter was sent to farmworker employers in the area telling them about the training. (In a couple of instances, farmers called us to express interest in the project. One grower sent a migrant family to the training.) Other planning elements of the program included selecting a date and a facility for the two trainings, recruiting participants, creating English and Spanish flyers to promote the program, and English and Spanish versions of participant applications. Bilingual consent forms were created for the parents of the participant youth to sign to allow the youth to participate in the training. We also created YouTube video consent forms for both adults and the parents of the youth participants. English and Spanish Consent forms were also developed and used when inviting the workshop participants to participate in a voluntary survey.

Each of the two workshops delivered similar content, consisting of seven CERT core sessions and the American Heart Association First Aid, CPR, and AED training. Extension educators also developed a session on money management in the event of an emergency. The trainer did not speak Spanish, so volunteer bilingual translators were recruited to assist participants who did not understand English. Volunteers also provided childcare. Most participants participated in the assessment of the workshops. The work group ended the fieldwork with the administration of an evaluation survey in June 2017, approximately a year after the June 2016 workshop.

Post training project achievements included the video tape production of the training and use of an album of video clips from the training to create 10 YouTube videos that featured participants. Among Extension’s many contributions to the project, these are available at https://www.youtube.com/channel/UCtzfWvjcdTzG-L-51bZpBlQ. The project leaders also conducted two nationwide webinars. The Chicago FEMA regional office learned about our program and FEMA in Washington D.C. invited us to feature our novel program to a national audience as part of its 2017 National African American History Month webinar entitled *Building Partnerships to Strengthen Preparedness through Diverse Communities* conducted on February 27, 2016 to 246 attendees. Among webinar participants were personnel of FEMA’s Individual and Community Preparedness Division, MSU Extension, MDHHS, and others. We also delivered an eXtension Foundation Zoom webinar on May 23, 2016, entitled *The Migrant and Seasonal Farmworker Disasters Preparedness Demonstration Project: Implementation and Lessons Learned*. Our purpose was to reach Extension and Migrant Health Center personnel, as well as others across the country. In addition, the PI delivered a Power Point presentation before the MDHHS Interagency Services Committee and a presentation before the Allegan/Ottawa/Barry Migrant Resource Council.

The use of the data and results of the project extend beyond YouTube productions, webinars, and conference and meeting presentations. The outreach, education, and research efforts associated with this program have produced case study methodology and evidence of program effectiveness. The project has also had a pedagogical effect. A recent session of the 2017, Michigan State University Extension Fall Conference by an Extension educator also used the YouTube videos from our program to share information about bilingual course development with MSU Extension personnel.

### Challenges

There were several challenges to implementing the disaster preparedness training to MSFWs in the field. Chief among them was to get the farmworkers to show up for the training. The staff of the Oceana MDHHS office recruited 38 farmworkers for the June training and eighteen for the October training, but only 14 showed up on day 1 of the June training and 11 the first day of the October training. The weather delayed the asparagus harvest until June, making it less of a priority for farmworkers to participate in the training, as many farmworkers had to work. Additionally, project-funding limitations prevented the project leaders from visiting farmworkers for recruitment. The project site was two hours away from the University, reducing the amount of times the project leaders could assist with recruitment. The staff of the Oceana MDHHS also had time constraints because it still had its work to do, in addition to helping with recruitment.

Not having a certified bilingual trainer available to conduct the training to a bilingual and monolingual audience presented a serious challenge. It moved the team to recruit MSFW teenagers to assist Spanish-only farmworkers in understanding the materials. However, the teenagers had their own issues learning the material, so they were less helpful than expected. Other Spanish speakers were recruited to assist Spanish-only workshop participants with understanding the training.

Another issue was the lack of transportation available for some of the workshop participants. Staff from the Oceana MDHHS office assisted with transporting farmworkers to the training site. There were some delays in transporting them, causing delays in the training, which was already tightly scheduled.

The curriculum used a “one-size fits all” approach, which failed to take into account the audience, which was quite diverse in age, English language skills, and other factors. Some participants questioned the relevancy of some of the topics. The content areas required for each module were quite broad, making it difficult to cover all the material comfortably in a three-day period. These time constraints also impacted the time available to conduct the evaluation.

### Evaluation summary

There is a gap in disaster preparedness and training for it in the Hispanic MSFW population. This project used the nationally recognized CERT curriculum to examine the feasibility and effectiveness of teaching farmworkers about disaster preparedness in receiving communities where they travel to work. In total, 22 MSFWs completed the training workshops and received both an AHA CPR Certificate and CERTs training certification to become members of the President’s Citizen Corps. An additional farmworker received only an AHA Heartsaver First Aid completion card. Nine were migrant farmworkers and twelve were seasonal farmworkers. Eight of the 22 who completed both training were under the age of 18. Another eight were between 19 and 29 years of age. Another three participants were between 30 and 39 years of age, and two were between forty and forty-nine years old. All but two participants were Hispanic. Eighteen of the 20 Hispanics were of Mexican origin. Two were Puerto Rican.

An assessment of the outcomes from a variety of sources suggests at least four types of impacts resulted from the intervention. First, survey evidence suggests participants benefited from the training as measured by their enhanced emergency response and disaster preparedness levels and their First Aid, CPR and AED competencies. The project team translated from English to Spanish a post-training survey instrument employed by Jane Peterson in previous trainings and used it to obtain feedback about the workshop and each of the training modules. This instrument has 13 questions on course evaluation and between 5 and 7 questions on the evaluation of each unit.

Participants evaluated the overall quality of the training highly. Although each of the units was assessed separately, here we highlight the course overall by looking at a few of the evaluation statements and how participants from post training surveys responded.

- The training was enjoyable and beneficial: 75% strongly agreed; 25% agreed.
- My knowledge and skills increased because of the training: 81% strongly agreed; 19% agreed.
- The materials distributed were helpful: 88% strongly agreed; 12% agreed.
- I would recommend this training to others: 94% strongly agreed; 6% agreed.

The workshop participants were not the only stakeholders to gain knowledge on disaster preparedness from participating in the project. A second beneficiary was the project team, who learned numerous valuable disaster preparedness training planning lessons. It learned, for example, that project planning and implementation both require partnerships and collaboration between the university and the relevant local stakeholders. Additionally, farmworkers’ work schedules, as well as the school schedules of the youth participants, needs to be considered when targeting an area and setting the times, duration, and structure of the training modules and workshops. Translating materials into Spanish is necessary for recruitment, training, and evaluation. Having a bilingual trainer is necessary.

A third set of disaster preparedness benefits from the project accrued to the receiving community where migrants travel to work. By completing the training, 30 participants became members of the President’s Citizen Corps and were certified in CPR, capable of serving a vital role in the interest of public health. Because some of those trained are seasonal farmworkers who reside in the area all year, there now exists a linguistic and cultural ability in the community to address the needs of this vulnerable population in the event of a disaster.

A fourth type of impact relates to evidence indicating growing interest by participants in continuing their emergency response training. If this pattern continues, it would enhance the capacity of the county, which is 15% Hispanic, to provide a bilingual and bicultural emergency response in the event of an emergency. An evaluation survey mailed to 24 participants (21 farmworkers and 3 former farmworkers) one year after the June 2016 training and nine months after the October training provides evidence of this project’s outcome. In response to the question, “Have you received additional disaster preparedness training since you took the course? Yes or No,” 8 respondents said yes and 16 said no. Of those who said yes, two had taken another CPR class at work and1had taken First Aid and CPR classes at work. Another participant took CPR and First Aid classes in high school. Additionally, five attended Oceana County CERT meetings/classes. The increased participation of farmworkers in these trainings implies a change in the ethnic and bilingual capacity of Oceana County CERT to respond to the needs of MSFWs in the area in the event of a disaster.

### Recommendations for refinement

Refinements to the training came from participant evaluation data as well as input from the project team. Among the key recommendations suggested by the participants were making the sessions bilingual, having shorter sessions over more days, and having more interactive group simulations.

The Mason-Oceana County MDHHS workgroup members recognized the recruitment and training challenges brought on by the nature of the MSFW population, but they attributed them to limitations in the program’s ability to adopt a more comprehensive recruiting approach. Although MDHHS staff was a great recruiting resource, the staff had responsibility of their own jobs, which at the peak of the harvest season put high demands on their time. The group recommended targeting labor camps in addition to employer and DHHS client lists. Other team members recommended targeting dairy workers who are in the area year round. However, time and funding restrictions prohibited adoption of this type of comprehensive recruitment effort.

Training would most likely have drawn more participants if the workshop sessions had not called on them to make a three-day commitment. Long-term sustainability of a disaster preparedness-training program in the area requires a local agency champion to sponsor the training on a regular basis. The training itself needs to be bilingual and flexible enough to work around the work schedules of the farmworkers.

The recruitment of farmworkers for the June workshop relied largely on the direct efforts of employees of the Michigan Department of Health and Human in Oceana County to recruit farmworkers for the training program. They would rely on client records and labor camp visits to recruit workshop participants. Program leaders and others also promoted the program through the network of human service agencies and churches. In addition, the project team sent letters to agricultural employers informing them of the training. Project team members also responded to phone calls from employers asking about the training. However, that was the extent of the outreach and marketing of the program to employers. The recruitment strategy for the October workshop was similar but supplemented by efforts by a local agency who committed to reaching out to local processing operations. However, project resource limitations prohibited the project leaders from developing and implementing a comprehensive recruitment strategy that also directly targeted the farm and processing plant employers of farmworkers in the area on a consistent basis.

### Future training and professional development needs

Michigan State University Extension personnel learned how to fit into the existing emergency response system in Oceana County in a unique way. Often, Extension personnel respond to the needs of agricultural and horticultural producers. The Extension Service can also provide information to the media in the event of a disaster [9]. In this case, Extension educators organized and facilitated the ability of the local emergency response system to take action in addressing the critically important needs of farmworkers to prepare for and respond to a disaster. Although the team relied on the Director of NCES to conduct the training, Extension personnel still had a vital role to play, serving as a catalyst in convening the relevant stakeholders in a community, and in planning, implementing, monitoring, and evaluating the education intervention.

The lack of bilingual FEMA-certified trainers in rural areas in northern states seriously hinders the ability to reduce the disaster preparedness training disparities that exist in the MSFW population in the places where they work. An important area of future research could address the inherent lack of bilingual-certified CERT trainers in rural areas and its impact on the ability to deliver CERT training to bilingual audiences. This is particularly relevant in communities that receive farmworkers to harvest the seasonal crops and do not have the capacity to serve this population in the event of a disaster. The lack of bilingual-certified trainers is a bottleneck that hinders the provision of disaster preparedness training for interested Spanish-speaking farmworker residents and migrants in the communities where they travel to work. It also hinders the capacity of the community to respond to the needs of this population in the event of a disaster emergency.

### Study limitations

Another project resource related constraint of this study had to do with the evaluation of the training program. One limitation was the potential for measurement error. Measurement error refers to how well or poorly a particular instrument performs in a given population [10]. Two of the three survey instruments used in this study were not piloted with the targeted population. The instruments were from existing CERT projects that were translated into Spanish for use in this study. Although this provided some benefits, this practice raises questions about the reliability and validity of the instrument, as well as its readability and clear understanding of meaning. To address this issue, survey administrators facilitated the survey process and tried to be as helpful as possible. However, it is unlikely this eliminated these limitations.

Another statistical limitation was the potential for selection bias due to the small sample size of the pilot study. There was a larger percentage of women and seasonal farmworkers in the study than the general farmworker population. Additionally, it is not possible to generalize the results of the pilot study to all Latino MSFWs. However, this was a pilot study to ensure the feasibility of the program. The results of this study should be tested with a greater number of participants and a more representative sample.

## Conclusion

This demonstration project was feasible due to the formation of a community/university partnership between MSU and the MDHHS, along with the use of culturally and linguistically appropriate recruiters and recruitment materials and the availability of volunteer translators and childcare services. Reaching recruitment goals was a challenge due to the weather, sufficient resources to recruit extensively using multiple strategies, and the lack of flexibility in training schedules. Although a bilingual-certified trainer was lacking, we relied on bilingual translators. The workshop participants were quite receptive to the training. The sessions were highly rated in terms of quality and usefulness. There was also evidence showing the training had an impact on the participants’ interest in continuing disaster preparedness training. If this pattern continues, there exists the potential to increase the community’s bilingual and bicultural capacity to respond to the needs of the MSFW population in the event of a disaster. The results support previously published research on the importance of linguistically and culturally relevant and practical approaches for the success of disaster preparedness training targeting minority populations [9–12].

The feasibility and sustainability of disaster preparedness education programs that target the MSFW population in rural areas where they travel to work hinges on having a local or state agency that champions MSFW disaster preparedness training. That agency needs to have the capacity and resources to deliver linguistically and culturally appropriate training, with the flexibility to accommodate the working needs of the participants. In Michigan, the Interagency Migrant Services Committee of the Michigan Department of Health and Human Services has taken up the issue to call for the this training and recently has had a meeting with the Michigan Farm Bureau to discuss the issue.

While training programs that prepare farmworkers to respond to emergencies are necessary for farmworkers in receiving communities where they travel to work, farmworker receiving communities also need to consider the training needs of their public health professionals and first responders. The *Migrant and Seasonal Farmworker Emergency Preparedness Guide* developed by the University of Chicago is an example of an excellent guide to help jurisdictions identify, plan for and operationalize their preparedness, respond and recovery efforts for this “at risk” population. Addressing such training needs are consistent with the national guidance and requirements of the Pandemic and All-Hazards Preparedness Reauthorization Act of 2013 that grants state health departments greatly needed flexibility in dedicating staff resources to meeting critical community needs in the event of a disaster.

## Abbreviations

AED: Automated external defibrillator
CERT: Community Emergency Response Team
CPR: cardiopulmonary resuscitation
EDED: Extension Disaster Education Network
FEMA: Federal Emergency Management Agency
MDHHS: Michigan Department of Health and Human Services
MSFW: Migrant and Seasonal Farmworker
MSFW-DPD: Migrant and Seasonal Farmworkers Disaster Preparedness Demonstration
MSU: Michigan State University
NCED: Newaygo County Emergency Services

## Footnotes

1 A survey of 300 farmworkers conducted in Oceana County in 2003 by the author indicated an average educational level of 6.8 years, some received in Mexico. Discussions with employees from the Oceana Department of Health and Human Services who work with MSFWs confirm low levels of education and a preference for communicating in Spanish.

2 The name is a pseudonym.
